# Graph-Based Semantic Web Service Composition for Healthcare Data Integration

**DOI:** 10.1155/2017/4271273

**Published:** 2017-08-20

**Authors:** Ngamnij Arch-int, Somjit Arch-int, Suphachoke Sonsilphong, Paweena Wanchai

**Affiliations:** ^1^Semantic Mining and Information Integration Laboratory (SMIIL), Department of Computer Science, Faculty of Science, Khon Kaen University, Khon Kaen 40002, Thailand; ^2^Graduate School, College of Asian Scholars, Khon Kaen 40000, Thailand

## Abstract

Within the numerous and heterogeneous web services offered through different sources, automatic web services composition is the most convenient method for building complex business processes that permit invocation of multiple existing atomic services. The current solutions in functional web services composition lack autonomous queries of semantic matches within the parameters of web services, which are necessary in the composition of large-scale related services. In this paper, we propose a graph-based Semantic Web Services composition system consisting of two subsystems: management time and run time. The management-time subsystem is responsible for dependency graph preparation in which a dependency graph of related services is generated automatically according to the proposed semantic matchmaking rules. The run-time subsystem is responsible for discovering the potential web services and nonredundant web services composition of a user's query using a graph-based searching algorithm. The proposed approach was applied to healthcare data integration in different health organizations and was evaluated according to two aspects: execution time measurement and correctness measurement.

## 1. Introduction

Web services (WS) composition is a method used to combine existing WS from heterogeneous systems to build more complicated business processes that match with user requirements. WS composition also accommodates the development of systems capable of automatic execution of multiple individual WS simultaneously [1]. In developing systems through WS composition, the most widely used business process execution languages that specify the services involved in the composition's execution environment are Web Services Business Process Execution Language (WS-BPEL) [2] or Web Service Choreography Interface (WSCI) [3]. However, these technologies do not offer well-defined semantic and expressive capability for solving semantic service discrepancies that occur due to disagreement in the meaning, interpretation, or intended use of service information. In most cases, this situation drives the challenge of creating an automated WS composition system that focuses on solving the problems of WS heterogeneities. These problems necessitate semantic matching of input and output parameters to combine multiple relevant services.

Richer semantics for WS provide greater automation of selection, composition, and invocation of heterogeneous services. Semantic Web Services (SWS) [4, 5] have emerged to facilitate automation and dynamism in WS discovery, selection, composition, and monitoring. SWS technologies such as Semantic Markup for Web Services (OWL-S) [6], Web Service Modeling Ontology (WSMO) [7], and Semantic Annotations for WSDL and XML Schema (SAWSDL) [8] have enabled well-known semantic representation languages for WS, which have prompted researchers to develop new WS composition techniques to automatically generate composite services. In recent decades, many approaches for WS composition have been proposed, and certain approaches, such as the work of Oh et al. [9], Hatzi et al. [10], Zou et al. [11], and Puttonen et al. [12], were aimed at fully automated WS composition using AI planning technology in which a sequence of actions is created from an initial state (inputs and preconditions) to a goal state (requested outputs). The plans that describe the sequence of WS actions to be executed are encoded in languages such as Planning Domain Definition Language (PDDL) [13, 14]. However, most of these proposals suffer from high complexity and time consumption for large-scale WS composition. Other approaches, such as the work of Rodriguez-Mier et al. [15] and Lin et al. [16], applied graph-based algorithms for WS composition to support efficient discovery and composition of large-scale WS in which a dependency graph model of WS is produced. However, such previous works did not present a method with which to prepare the WS dependency graph and further necessitated the difficult task of manually updating the graph. Although the work of Yue et al. [17] and Shin et al. [18] further proposed a graph preparation approach that constructs the graph model of related services automatically via a syntactic matching technique and functional WS semantics for determining the association among services, the semantic conflict problems within the WS parameters still exist when handling multiple WS through different sources.

In this paper, we propose a graph-based SWS composition system and introduce a dependency graph preparation approach that aims to resolve the problem of semantic discrepancies through the use of semantic matchmaking rules to automatically generate the WS dependency graph. The proposed approach enables the inference engine to perform flexible semantic matches to create a graph model of the related services. This approach is capable of supporting scalable data within graph model by storing it in a corresponding graph database. We further propose a nonredundant WS composition approach that can efficiently search the most satisfactory services for customer queries using a dependency graph search technique. To ensure that the proposed approaches can be applied in practical settings, we have developed web-based applications consisting of the graph management tools and WS composition search engine, which are necessary for discovery and publication of complex services in a healthcare domain. Additionally, our proposed approaches are evaluated according to two aspects: the execution time measurement and the correctness measurement.

The remainder of the paper is structured as follows.  Section 2 presents a review of the related literature, Section 3 outlines the proposed system architecture, Section 4 offers motivating examples, Section 5 presents the graph-based SWS composition methodology of the proposed system, Section 6 illustrates the system implementation, Section 7 presents a system evaluation and discusses the contributions and makes comparison with other works, and conclusions and recommendation for future work are summarized in Section 8.

## 2. Literature Review

WS composition enables achievement of particular goals through a process of primitive controls and exchanges. This concept leads to the development of numerous technologies, such as WS-BPEL [2] and WSCI [3], which create the ability to integrate distributed WS into a business process (or process model). The exploitation of WS-BPEL, as presented by Chao et al. [19], Curbera et al. [20], Huang et al. [21], Lee et al. [22], and Yu et al. [23], promises to facilitate business transactions across different companies. However, WS-BPEL is based on the XML standard, which lacks the necessary support in semantic annotation required to solve semantic discrepancies involving dynamic WS composition.

The objective in promoting the SWS is to create a flexible layer for development of an automatic system with dynamic discovery, composition, and execution of WS [4]. The common infrastructure of SWS involves specification of semantic annotation combined with WS standards, that is, eXtensible Markup Language (XML), Simple Object Access Protocol (SOAP), Web Services Description Language (WSDL), and Universal Description, Discovery, and Integration (UDDI). Following the semantic WS annotation reviews of Tosi and Morasca [24], one of the semantic annotation languages for WS is OWL-S [6], which represents the profiles, process model, and grounding of WS through the Web Ontology Language (OWL) [25]. The use of such semantic descriptions enables a more flexible and expressive capability for discovery, composition, and execution of WS. Many research works have aimed at techniques of discovering, composing, or developing services as reviewed in Rao and Su [26], Lemos et al. [27], and Rodriguez et al. [28]. The need still exists for automatic WS composition to solve the problems within various domains.

Many research efforts have been conducted in automatic WS composition using different techniques. In the context of the AI planning technique, the work of Hatzi et al. [10] presented an integrated approach for SWS composition by exploiting AI planning techniques. The approach is based on transforming the WS composition problem into a planning problem that is encoded in PDDL and solved by external planners. The produced composite services are transformed back to OWL-S. The work of Zou et al. [11] considered the WS composition problem as a WS composition planning problem and used AI planners to find a composition plan for the composition request. The available services are converted into a planning domain in PDDL and translate a composition request into a planning problem in PDDL. A WS composition planning problem is subsequently fed into an AI planner to automatically find a composition plan corresponding to the given composition request. The work of Puttonen et al. [12] presented a web-service-based framework capable of automatically composing WS applying to the factory automation domain. The framework aims to extract the planning actions from the OWL-S service descriptions and create a mapping from each action to convert the acquired solution plans into composite OWL-S processes. The results are intended to reduce the workload of developing semantic WS descriptions and enable automatic composition and deployment of workflow descriptions. Through logic-based technique and algorithms, the work of Rao et al. [29] proposed an automated composition of SWS using the Logical Linear (LL) theorem to prove the rules required for extracting the compositions of WS. The work of Kwon and Lee [30] proposed a nonredundant WS composition approach based on a two-phase algorithm capable of efficient searching of the scalable WS data using the relational database indexing technique.

In the context of a semantic-based technique, the work of Kona et al. [31] proposed the semantic matching techniques to arrange WS from the repository in which the input and output are semantically matched. The work of Talantikite et al. [32] developed a model of semantic annotation for WS discovery and composition using the ontology-based similarity measurement between concepts. The work of García-Sánchez [33] proposed an agent-based framework for service integration and interoperability in an e-Government domain, in which the discovery agent is represented for matchmaking and composing services through the SWS and ontology concepts mapping. Another work was proposed by Bansal et al. [34], who presented a generalized semantics-based technique for automatic service composition.

Within the graph-based technique, models of WS composition have been proposed in several research studies. The work of Hashemian and Mavaddat [35] proposed an original approach that used a graph search algorithm for WS composition with functional capability. The work of Dong-Hoon and Kyong-Ho [36] proposed an accurate WS composition approach by enhancing the functional semantic consideration in graph searching. The work of Ukey et al. [37] proposed a model of WS composition based on a bidirectional heuristic algorithm working in tandem with the WS dependency graph. The work of Wang et al. [38] studied the problem of finding the minimum cost service composition (MCSC) for a general service composition request, which is represented by a directed acyclic graph (DAG). The work of Rodriguez-Mier et al. [15] proposed an automatic WS composition technique based on a heuristic-based graph search algorithm. The work of Lin et al. [16] proposed a cost-effective planning graph approach based on a backward strategy for large-scale WS composition in a cloud environment. This work aims to design a cost-effective WS composition algorithm to obtain multiple service compositions using fewer numbers of WS at low costs and within an acceptable execution time. Finally, the work of Shin et al. [18] proposed a graph-based composition method that uses the functional semantics of WS and a representative action to represent the actions of a service. This approach uses an AND/OR graph to store data dependencies for WS composition, and rules are created to map a service and combine service actions.

The main contributions of this paper and a comparison of our WS composition approach with the other approaches will be discussed in Section 7.

## 3. Proposed System Architecture Overview

**Figure 1:**
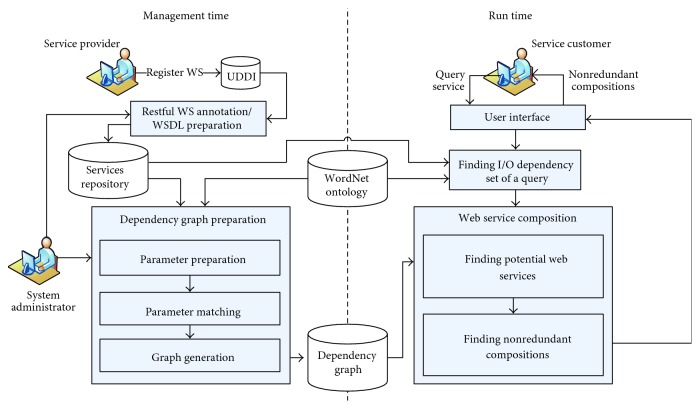
Architecture of graph-based Semantic Web Services composition system.

This section presents an overview of the graph-based SWS composition system, which is divided into two subsystems of management time and run time, as illustrated in Figure 1.

1. The management-time subsystem: This subsystem is designed to maintain the data for preparation of the WS dependency graph. The system administrator can annotate the RESTful WS or import the WSDL registered in the UDDI registry (RESTful WS annotation/WSDL preparation step) in which the service is parsed and stored in Resource Description Framework (RDF) format within the services repository. The services repository contains the labeled functional capability of WS, including the service and operation names, input, and output. These initial data are used in the dependency graph preparation process. This process consists of the following three steps:
  - Parameter preparation: The name of each input (or output) parameter of WS is tokenized into keywords in the parameter preparation process.
  - Parameter matching: In this step, the keywords are used to compare other parameters using the semantic matchmaking rules in the parameter matching process with the aid of WordNet [39], which is a lexical database of English providing the relationship between senses of words. These words' senses (synsets) are sets of cognitive synonyms expressing a distinct concept and interlinking with other senses by means of conceptual semantic.
  - Graph generation: The calculated matching coefficients of each pair of parameters are used to generate the dependency graph of WS through a graph generation rule.
2. The run-time subsystem: This subsystem creates a user interface in which the service customers can pose a query to search the services through keywords. The requested keywords are used to find the I/O dependency set of matched input and output parameters in the services repository through the WordNet similarity measurement. This process consists of the following two steps.
  - Finding potential web services: The I/O dependency set is used to find the potential WS paths (subgraphs) from the dependency graph model of WS with the use of a graph search algorithm.
  - Finding nonredundant compositions: These paths are constructed as nonredundant WS composition (composite processes) through the proposed algorithm. The obtained results of the nonredundant WS composition can be exported to standard executable languages, such as OWL-S or BPEL4WS, which might be further used in creating the WS invocation.

The details of the processes in management-time and run-time subsystems are described in the dependency graph preparation and WS composition subsections of Section 5, respectively.

## 4. Motivating Example

This section illustrates an example of WS as shown in Table 1. These services extended from our previous work [40, 41] and were developed for retrieval of healthcare data from heterogeneous Electronic Health Record (EHR) systems of different health organizations. The operations were created in both SOAP WS and RESTful WS versions, which return healthcare data in XML format. The example consists of eight operations of WS (i.e., *PI*, *PD*, *PHI*, *DS*, *DXI*, *OI*, *DI*, and *ZI*) and service requests (*q1* and *q2*). Given a query *q1*, the requested input of *q1* is citizen-id, and the requested outputs of *q1* are *health-number*, *organization-name*, *district-name*, and *zip-code*. Although, the *PI* and *PD* are services that satisfy the input of *q1*, the output of *PI* and *PD* does not fully satisfy all requested outputs of *q1*. Certain requested outputs of *q1*, such as the *organization-name*, *district-name*, and *zip-code*, are satisfied in the operations *OI*, *DI*, and *ZI*, respectively. The operations *PI* and *PD* return the outputs as *hospital-code* and *district-code*, which might be used as inputs of *OI*, *DI*, and *ZI*. Thus, the composition of WS {*PI*, *PD*}→{*OI*, *DI*, *ZI*} should satisfy a query *q1*, which can be presented to the service customer as two nonredundant compositions: {*PI*}→{*OI*, *DI*, *ZI*} and {*PD*}→{*OI*, *DI*, *ZI*}. Although the semantic matchmaking techniques of WS composition are limited to small-scale WS, this research proposes graph-based search algorithms to efficiently find a nonredundant composition of WS with a large-scale WS. The research also creates a systematic method of WS dependency graph preparation to enable the inference engine to perform semantic matching between the input and output parameters of WS.

## 5. A Graph-Based Semantic Web Services Composition Methodology

This section presents the definition of semantic parameter matching and the components of a graph-based WS composition ontology, which are used in two main processes of the proposed system, that is, dependency graph preparation and WS composition. These processes are described as follows.

### 5.1. Semantic Parameter Matching Definition

According to the semantic matchmaking technique of WS capabilities designed based on the ontology concepts, the matching types can be classified into several levels, such as Exact, Plug-in, Subsumes, and Fail [42]. This research considers only three matching types of Exact, Subsumes, and Fail, (as defined in Table 2) for use in parameter matching of dependency graph preparation in the next section.

### 5.2. Graph-Based Semantic Web Services Composition Ontology

**Figure 2:**
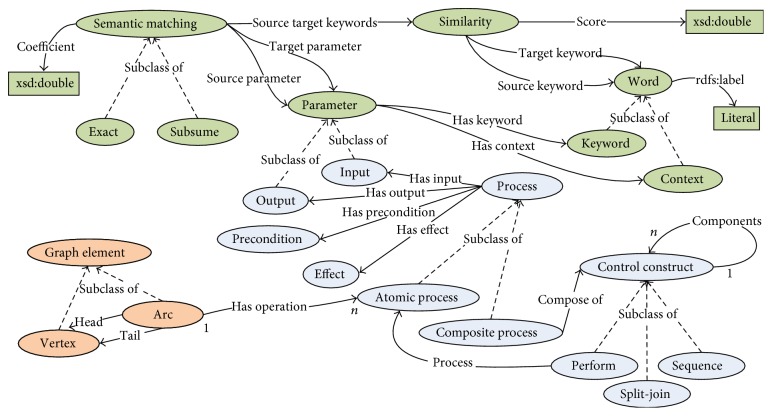
Structure of a graph-based Semantic Web Services composition ontology.

In this section, we propose the graph-based SWS composition ontology, which is used to represent the components of WS in the dependency graph preparation. A portion of the graph-based ontology, which is also expressed in OWL language, is illustrated in Figure 2. The proposed ontology consists of three main classes: *GraphElement*, *Process*, and *SemanticMatching*. The *GraphElement* class, which consists of the *Arc* and *Vertex* as subclasses, is derived from the directed graph theory and is used to describe the dependency of services. The *Arc* class represents redundant operations that have the same functions through the property *hasOperation* and describes the input and output through the *tail* and *head* properties, respectively. The *Vertex* class is defined to represent input or output parameters of operations represented in *Arc*. The *Process* class, which is derived from the OWL-S process model specification [43], describes (1) the characteristics of the *AtomicProcess* and the *CompositeProcess* classes through *hasInput*, *hasOutput*, *hasPrecondition*, and *hasEffect* properties and (2) the control constructs (*Perform* and *Sequence*) of the *CompositeProcess* class. The *SemanticMatching* class describes the semantic similarity of parameters through the *sourceParameter* and *targetParameter* properties, which are classified into two types of *Exact* and *Subsume*, as defined in Table 2. This graph-based SWS composition ontology is used in the dependency graph preparation in the next section.

### 5.3. Dependency Graph Preparation

The dependency graph preparation is the process of constructing the relationships of the input and output parameters of the atomic process as a graph. This process consists of three interrelated processes of parameter preparation, parameter matching, and graph generation processes, as described in the following subsections.

#### 5.3.1. Parameter Preparation

Parameter preparation is a process used to divide the parameter names of the atomic processes into meaningful keywords. For this process, the basic components of the graph-based SWS composition ontology, such as *AtomicProcess*, *Parameter*, *Keyword*, and *Context*, are defined as sets of atomic processes of WS, input and output parameters of atomic processes, keywords of parameters, and contexts of parameters, respectively. Each basic component is used in the following subprocesses.

1. *Creating Context*. This process is used to create a context *t*_*c*_ ∈ *Context* of a parameter *p*_*c*_ ∈ *Parameter* by adjusting the format of a parameter *p*_*c*_ according to the following rules:
  Rule 1. If the name of *p*_*c*_ contains special characters such as plus and minus, these characters are replaced by the delimiter symbol “_” (underscore).
  Rule 2. If the name of *p*_*c*_ matches the regular expressions of capitalized words, the delimiter “_” is inserted between words.
  Rule 3. If the name of *p*_*c*_ contains prepositions such as “of” and “with” or articles such as “a,” “an,” and “the,” these characters are removed from the parameter.

**Example 1.**

**Figure 3:**
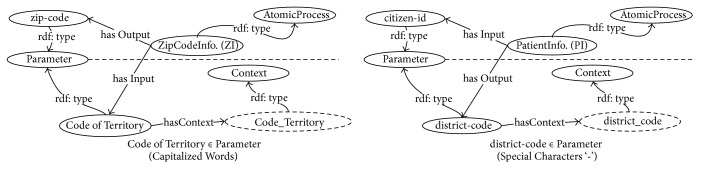
Example of the context creation presented in graph-based SWS composition ontology.

Given the {“*CodeOfTerritory*”, “*district-code*”} ∈ *Parameter* are input and output parameters of {*ZI*, *PI*} ∈ *AtomicProcess*, respectively. Following Rules 1–3, the string “CodeOfTerritory” is added with a delimiter “_” between words beginning with capitalized character (resulting in “Code_Of_Territory”), and the prepositions “Of” are removed following Rule 3 (resulting in “Code_Territory”). In the next parameter, “district-code” is replaced by the delimiter “_” following Rule 1 (resulting in “district_code”). Finally, {“*Code_Territory*”, “*destrict_code*”} ∈ *Context* are constructed as contexts of parameters {“*CodeOfTerritry*”, “*district-code*”} ∈ *Parameter*, respectively. The execution results of Rules 1–3 for Example 1 are further presented in a portion of the graph-based SWS composition ontology, as illustrated in Figure 3.

(2) *Creating Keyword*. This process is used to create a keyword *k*_*c*_ ∈ *Keyword* of a parameter *p*_*c*_ ∈ *Parameter* by extraction from a context *t*_*c*_ ∈ *Context* of a parameter *p*_*c*_ according to the following rules:
  Rule 4. If a context *t*_*c*_ contains a delimiter symbol, a substring before the first delimiter symbol of *t*_*c*_ is adjusted to a lowercase substring and extracted as a keyword of *p*_*c*_, and the substring after the delimiter symbol is assigned as a new context of *p*_*c*_.
  Rule 5. If a context *t*_*c*_ does not contain a delimiter symbol, *t*_*c*_ is removed and constructed as a lowercase keyword of *p*_*c*_.

**Example 2.**

**Figure 4:**
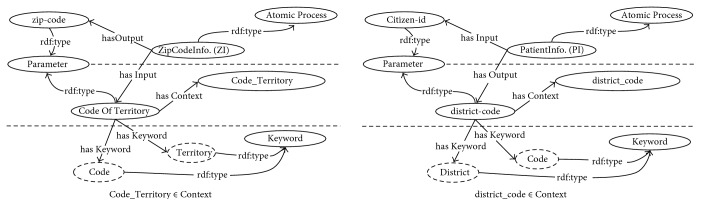
Example of the keyword creation reasoning result.

Following the parameters of Example 1, the “*CodeOfTerritory*” parameter can be created in this context as “*Code_Territory*”. After performing the first round of Rule 4, the lowercase string “*code*” is extracted as a first keyword, and the string “*Territory*” is constructed as the new context. The context string “*Territory*” is removed and reconstructed as the lowercase keyword “*territory*”, according to Rule 5. Hence, the {“*code*”, “*territory*”} ∈ *Keyword* is a set of keywords of the parameter “*CodeOfTerritory*”. For the next context, “*district_code*” is extracted to {“*district*”, “*code*”} ∈ *Keyword* of the parameter “*district-code*”. The results of execution of Rules 4-5 for Example 2 are illustrated in Figure 4.

#### 5.3.2. Parameter Matching

Parameter matching is the process of locating the semantic similarity between pairs of input and output parameters in WS operations. The matching instances are subsequently created as outputs. The parameter matching processes consists of keyword matching and matching filtering processes, as described below.

1. *Keyword Matching*. The keyword matching process calculates the similarity between keywords for each parameter using the WordNet database. Similarities between a pair of words are used to generate the matching instances between parameters.

Let {*ap*_*c*_, *ap*_*k*_} ∈ *AtomicProcess* be set of a pair of atomic processes or operations of WS. Let {*p*_*cm*_, *p*_*kn*_} ∈ *Parameter* be set of a pair of output and input parameters of {*ap*_*c*_, *ap*_*k*_}, respectively. Let *K*_*cm*_ and *K*_*kn*_ be sets of keywords of parameter *p*_*cm*_, and *p*_*kn*_, respectively. To calculate the similarity value between keywords, we apply the equation proposed by Wu and Palmer (wup) [44], which is defined in the following function.

*Sim*_*wup*_ : *K*_*cm*_ × *K*_*kn*_ → *V*, where *V* = {*v*_*i*_  | ∀*i* = 1,…, *n*  and  0 ≤ *v*_*i*_ ≤ 1, with *v*_*i*_ as the similarity value}.

The semantic similarity between keywords *k*′_*cm*_ ∈ *K*_*cm*_ and *k*′_*kn*_ ∈ *K*_*kn*_ is determined using the degree of similarity score (0,…, 1) calculated through the function presented above. If *Sim*_*wup*_(*k*′_*cm*_, *k*′_*kn*_) is equal to 1, these two keywords are an *exact match* that means they are synonymous. Otherwise, the subsume match can be determined through the adjustable threshold of semantic similarity degree. In this paper, the threshold of *subsume match* is set as 0.7 to determine subsumption relationship among closely related parameters corresponding to our previous studies on ontology mapping technique [45]. The semantic matching between parameters can be performed using the following rules:

Rule 6. (Exact match) if *Sim*_*wup*_(*k*′_*cm*_, *k*′_*kn*_) = 1, denoted by *k*′_*cm*_≅*k*′_*kn*_, where *k*′_*cm*_ ∈ *K*_*cm*_, *k*′_*kn*_ ∈ *K*_*kn*_, then an exact match instance *e*_*mn*_ ∈ *Exact* of the parameter pair (*p*_*cm*_, *p*_*kn*_) is created. The instance *e*_*mn*_ has source and target parameters of *p*_*cm*_ and *p*_*kn*_ and has the instance *sim*_*cmc*−*knk*_ ∈ *Similarity* containing the source-target keywords similarity.
Rule 7. (Subsume match) if 1 > *Sim*_*wup*_(*k*′_*cm*_, *k*′_*kn*_) ≥ 0.7 and *k*′_*cm*_⊆*k*′_*kn*_ or *k*′_*kn*_⊆*k*′_*cm*_, where *k*′_*cm*_ ∈ *K*_*cm*_ and *k*′_*kn*_ ∈ *K*_*kn*_, then a subsume match instance *ss*_*mn*_ ∈ *Subsume* of the parameter pair (*p*_*cm*_, *p*_*kn*_) is created. The instance *ss*_*mn*_ has source and target parameters of *p*_*cm*_ and *p*_*kn*_ and has the instance *sim*_*cmc*−*knk*_ ∈ *Similarity* containing the source-target keywords similarity.

**Example 3.**

**Figure 5:**
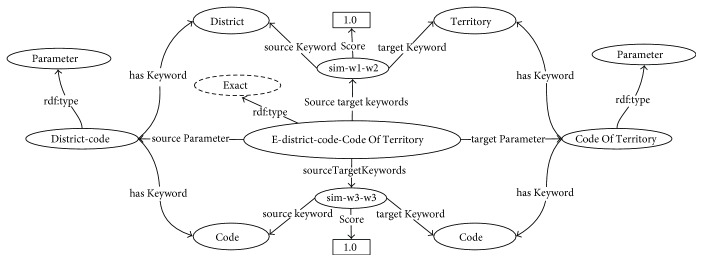
Example of the generated exact matching instance.

Following the procedure outlined in Example 2, a set of a pair of output and input parameters {*district-code*, *CodeOfTerritory*} ∈ *Parameter* of the atomic processes {*PI*, *ZI*}, respectively, contains the similarity scores between keywords as *Sim_wup_*(*district*,*territory*) = 1 and *Sim_wup_*(*code*,*code*) = 1. Hence, the *district* ≅ *territory* and *code* ≅ *code*. Thus, the instance *E-district-code-CodeOfTerritory* ∈ *Exact* is created. This instance has the *district-code* and *CodeOfTerritory* as the source and target parameters, respectively, and has two pairs of equivalent source-target keywords: *sim-district-territory* ∈ *Similarity* and *sim-code-code* ∈ *Similarity*. An example of this exact matching instance generated according to Rule 6 is illustrated in Figure 5.

**Example 4.**

**Figure 6:**
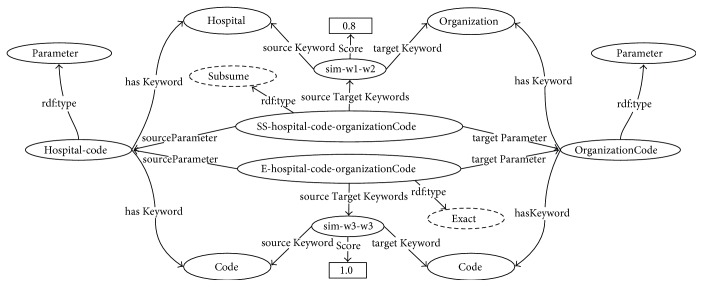
Example of the generated subsume and exact matching instances.

A set of a pair of output and input parameters {*hospital-code*, *organizationCode*} ∈ *Parameter* of the atomic processes {*PI*, *OI*}, respectively, contains the similarity scores between keywords as *Sim_wup_*(*hospital*, *organization*) = 0.8 and *Sim_wup_*(*code*, *code*) = 1 and *hospital* ⊆ *organization*. Hence, the matching instances *SS-hospital-code-organizationCode* ∈ *Subsume* and *E-hospital-code-organizationCode* ∈ *Exact* are generated, which have a pair of *hospital-code* and *organizationCode* as the source and target parameters. The instance *SS-hospital-code-organizationCode* has similar source-target keywords as *sim-hospital-organization* ∈ *Similarity*, whereas the instance E-hospital-code-organizationCode has similar source-target keywords as *sim-code-code* ∈ *Similarity*. An example of these matching instances generated according to Rules 6 and 7 is illustrated in Figure 6. Although this example contains both subsume and exact instances, one of these matching instances is eliminated through the matching filtering process described in the next section.

(2) *Matching Filtering*. The matching filtering process calculates the coefficient of the generated parameter matching instances of the keyword matching process to eliminate the irrelevant matching instances of the parameter pairs.

Let {*ss*_*ck*_, *e*_*ck*_} ∈ *SemanticMatching* be subsume and exact matching instances, respectively, between *p*_*cm*_ and *p*_*kn*_ where {*p*_*cm*_, *p*_*kn*_} ∈ *Parameter*. The coefficient of matching between *p*_*cm*_ and *p*_*kn*_, denoted as *Co*_*jac*_(*p*_*cm*_, *p*_*kn*_), is calculated according to Jaccard's coefficient [46], as shown in the following equation:

(1) $$\begin{array}{c} {Co}_{jac}\left(p_{cm},p_{kn}\right)=\frac{p}{p+q+r}, \end{array}$$

where *p* is the number of exact or subsume match instances found between *p*_*cm*_ and *p*_*kn*_, *q* is the number of keywords in *p*_*cm*_ that cannot be matched with any keyword in *p*_*kn*_, and *r* is the number of keywords in *p*_*kn*_ that cannot be matched with any keyword in *p*_*cm*_.

The return value of (1) is lowest if *p* is equal to 0 and is the highest if *q* and *r* are both equal to 0. Thus, the values of *Co*_*jac*_(*p*_*cm*_, *p*_*kn*_) lie in the range of (0,…, 1). According to the coefficient value of each match, the matching filtering is performed with the following rules:

Rule 8. If *Co*_*jac*_(*p*_*cm*_, *p*_*kn*_) = 1 and there exists only *ss*_*ck*_ ∈ *Subsume* or *e*_*ck*_ ∈ *Exact* between *p*_*cm*_ and *p*_*kn*_, then *ss*_*ck*_ or *e*_*ck*_ is retained. The finalized matching degree between *p*_*cm*_ and *p*_*kn*_ is either an exact match or a subsume match.
Rule 9. If *Co*_*jac*_(*p*_*cm*_, *p*_*kn*_) = 1, and there exist both *ss*_*ck*_ ∈ *Subsume* and *e*_*ck*_ ∈ *Exact* between *p*_*cm*_ and *p*_*kn*_, then *ss*_*ck*_, is retained, and *e*_*ck*_ is removed. The finalized matching degree between *p*_*cm*_ and *p*_*kn*_ is subsume match.
Rule 10. If *Co*_*jac*_(*p*_*cm*_, *p*_*kn*_) < 1, then all generated matching instances (*ss*_*ck*_ ∈ *Subsume* and *e*_*ck*_ ∈ *Exact*) between *p*_*cm*_ and *p*_*kn*_ are removed. The finalized matching degree between *p*_*cm*_ and *p*_*kn*_ is fail.

**Example 5.**

Following the generated matching instance of Example 3, the *E-district-code-CodeOfTerritory* ∈ *Exact*, which is only a matching instance between parameters *district-code* and *CodeOfTerritory*, has two pairs of equivalent keywords (*p* = 2). A number of keywords that are only positive for *district-code* are equal to zero, whereas a number of keywords that are only positive for *CodeOfTerritory* are also equal to zero (*q* and *r* = 0). According to Rule 8, the *Co_jac_*(*district-code*,*CodeOfTerritory*) = 1, and there exists only an exact instance, and thus the matching degree between parameters *district-code* and *CodeOfTerritory* is an exact match.

**Example 6.**

Following the generated matching instance of Example 4, the SS-hospital-code-organizationCode ∈ *Subsume* and *E-hospital-code-organizationCode* ∈ *Exact* are generated as two matching instances of a pair of parameters (*hospital-code*, *organizationCode*). According to Rule 9, the coefficient *Co_jac_*(*hospital-code*,*organizationCode*) = 1; there exist both subsume and exact match instances, and the exact match instance is removed. Hence, the matching degree between parameters *hospital-code* and *organizationCode* is a subsume match.

Examples of filtering results of the parameter matching instances are shown in Table 3.

#### 5.3.3. Graph Generation

Graph generation is the process of construction of a dependency graph model of WS in the services repository. The rules for reasoning of the dependency graph are given in Rules 11 and 12 as follows:

Rule 11. Let *ap*_*c*_ ∈ *AtomicProcess* denote an operation of WS. Let {(*i*_*ci*_, *o*_*cj*_) ∣ *i*_*ci*_ ∈ *Input* and *o*_*cj*_ ∈ *Output*} be a set of a pair of input and output of the operation *ap*_*c*_. The arc *a*_*c*_ ∈ *Arc* generates the parameters *i*_*ci*_ and *o*_*cj*_ ∈ *Vertex* as tail and head vertices, respectively. The arc *a*_*c*_ has the property *hasOperation with ap*_*c*_ as a property value.
Rule 12. Let {*ap*_*c*_, *ap*_*k*_} ∈ *AtomicProcess* denote a set of two operations of WS. Let {(*i*_*cm*_, *o*_*cn*_), (*i*_*km*_, *o*_*kn*_)} be a set of a pair of input and output parameters of *ap*_*c*_ and *ap*_*k*_, respectively, where *i*_*cm*_, *i*_*km*_ ∈ *Input* and *o*_*cn*_, *o*_*kn*_ ∈ *Output*. If there exists a matching instance *m*_*c*_ ∈ *SemanticMatching* between *o*_*cn*_ and *i*_*km*_, then arc *a*_*c*_ ∈ *Arc* is created. The arc *a*_*c*_ generates the parameters *i*_*cm*_, *i*_*km*_ ∈ *Vertex* as tail and head vertices, respectively. The arc *a*_*c*_ has the property *hasOperation* with *ap*_*c*_ as a property value. The implementation of this rule is described in Table 4.

**Example 7.**

**Figure 7:**
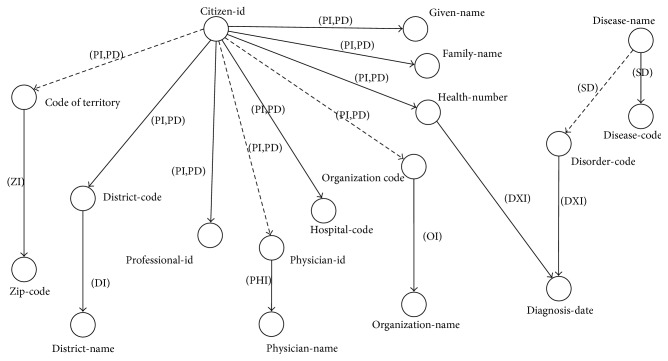
Example of the dependency graph of WS generated from Rules 11 and 12 executions.

According to the services description presented in Table 1, {*PI*, *ZI*} ∈ *AtomicProcess* has a set of a pair of input and output parameters as {(*citizen-id*, *district-code*), (*CodeOfTerritory*, *zip-code*)}, respectively. Because there exists a matching instance *E-district-code-CodeOfTerritory* of *district-code* and *CodeOfTerritory* parameters, even if *PI* (and *PD*) has no output represented as *CodeOfTerritory*, the arc *citizen-id-CodeOfTerritory* ∈ *Arc* (with assigned redundant operation set {*PI*, *PD*}) is generated. The complete result of the dependency graph of WS generated from Rule 11 and Rule 12 is illustrated in Figure 7.

### 5.4. Web Service Composition

WS composition is the process of finding the composite processes that satisfy a query from the service customer. This process consists of two interrelated steps: finding the potential WS and finding the nonredundant compositions.

#### 5.4.1. Finding Potential Web Services

The first step of the approach is searching the WS in the graph-based WS repository that might satisfy the composition. This step consists of two phases of algorithms: forward search and backward search algorithms. Let *q* denote the query of a service customer, *I*_*q*_ = {*i*_*qi*_ | ∀*i* = 1,…, *n*} be a set of input of *q*, and *O*_*q*_ = {*o*_*qi*_ | ∀*i* = 1,…, *n*} be a set of output of *q*. The I/O dependency set of *q* is given as a set of *D*_*q*_ = *I*_*q*_ × *O*_*q*_. Let *d*_*q*_ = (*i*_*qc*_, *o*_*qk*_) ∈ *D*_*q*_. The system performs a forward graph search through Algorithm 1 with inputs (*i*_*qc*_, *o*_*qk*_) and returns a potential WS path of dependency *d*_*q*_ as output.

**Example 8.**

**Figure 8:**
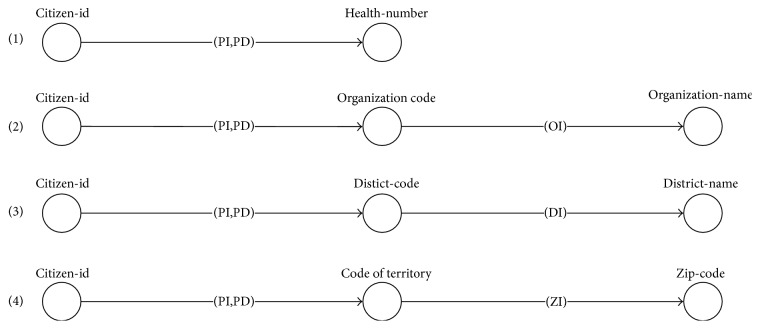
Example of path search results through Algorithm 1.

According to the example of WS and queries (Table 1), a query *q1* has I/O dependency (with semantic matching) found as set *D_q1_* = {(*citizen-id*, *health-number*), (*citizen-id*, *organization-name*), (*citizen-id*, *district-name*), (*citizen-id*, *zip-code*)}. Each I/O dependency in *D*_*q*1_ is used to find the potential WS path through Algorithm 1 and generate the result path sets (paths 1–4), as illustrated in Figure 8.

To obtain all WS paths for all inputs and outputs of the service request, the backward search is performed after the forward search through Algorithm 2. This algorithm aims to discover the potential WS paths that have output corresponding to the requested service's output but might have input that differs from the requested service's input. The discovered paths from both the forward and backward searches are combined as illustrated in the next section.

**Example 9.**

**Figure 9:**
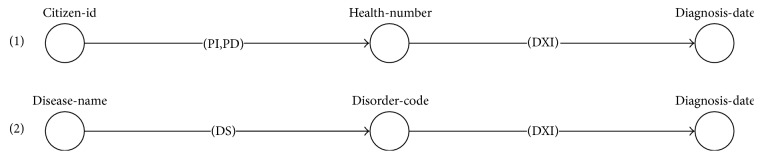
Example of path search results through backward search of Algorithm 2.

A query *q2* has I/O dependency set as *D_q2_* = {(*citizen-id*, *diagnosis-date*)}, according to the example of WS and queries (Table 1). The I/O dependency *D*_*q*2_ is used to find the potential WS paths through Algorithm 2, as illustrated in Figure 9.

#### 5.4.2. Finding Nonredundant Compositions

After fulfilling the potential WS paths in the dependency graph corresponding to the service query, the second step finds the nonredundant compositions from the potential WS paths. Let *Pt*_*q*_ = {*pt*_*i*_ ∈ *Pt*_*q*_ | ∀*i* = 1,…, *n*} be a set of the result paths of *D*_*q*_.  Algorithm 3 receives *pt*_*c*_ ∈ *Pt*_*q*_ as an input and returns a set of nonredundant composition paths.

**Example 10.**

**Figure 10:**
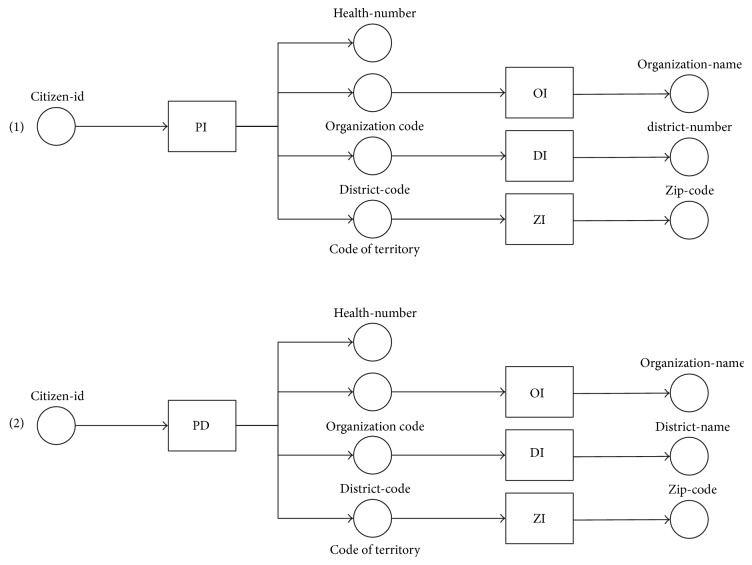
Results of nonredundant compositions (composite processes) of obtaining patient information.

Following the obtained results from Example 8, let *Pt*_*q*1_ = {*pt*_1_, *pt*_2_, *pt*_3_, *pt*_4_} be a set of potential WS paths of a query *q1*.  Algorithm 3 separates the redundant operations represented in the arcs of each path. For instance (path 2 in Figure 8), the first arc of *pt_2_* has a redundant operation set *RO*_1_, where {*PI*, *PD*} ∈ *RO*_1_. The operations *PI* and *PD* are separated into *cp*_1_, *cp*_2_ ∈ *CompositeProcess* because they have the same function (see descriptions in Table 1). Consequently, the next arc of *pt_2_* containing the operation {*OI*} is constructed as a sequential operation of *PI* and *PD* in the *cp*_1_ and *cp*_2_, respectively. Additionally, the nonredundant compositions returned from each *pt*_*i*_ ∈ *Pt*_*q*1_ are combined into more complex composite process constructs, such as sequential (*Sequence*) and parallel (*Split-Join*). An example of the complete results of the nonredundant WS composition from *Pt*_*q*1_ is shown in Figure 10.

**Example 11.**

**Figure 11:**
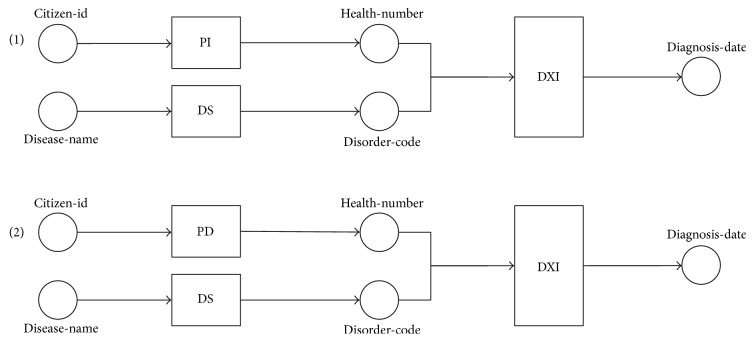
Results of nonredundant composite processes of obtaining diagnosis information.

Following the obtained results from Example 9, let *Pt*_*q*2_ = {*pt*_1_, *pt*_2_} be a set of potential WS paths.  Algorithm 3 separates the redundant operations represented in the arcs of each path. For instance (path 1 in Figure 9), the first arc of *pt*_1_ has a redundant operation set *RO*_1_ where {*PI*, *PD*} ∈ *RO*_1_. The operations *PI* and *PD* are separated into *cp*_1_, *cp*_2_ ∈ *CompositeProcess* because they have the same function (see descriptions in Table 1). Consequently, the next arc of *pt_1_* containing the operation {*DXI*} is constructed as a sequential operation of *PI* and *PD* in the *cp*_1_ and *cp*_2_, respectively. The nonredundant compositions returned from each *pt*_*i*_ ∈ *Pt*_*q*2_ can be combined into more complex composite process constructs, as shown in Figure 11.

## 6. System Implementation

This section presents implementation of the graph-based SWS composition system supporting the WS discovery process, which can satisfy combinations of services from the basic atomic services. To ensure the viability of model realization, we developed the SWS composition system for the healthcare institutes located in the northeast of Thailand, which include provincial hospitals, community hospitals, and primary healthcare units. Each healthcare institute offers database access services from different EHR systems. The SWS composition system was developed as a web application based on the Java platform. According to the proposed system architecture in Section 3, the system interfaces are presented into two subsections as follows.

### 6.1. The Management-Time Subsystem

For the management-time subsystem of the system architecture, the administrator application (http://202.28.94.50/wscomposition/admin) has been developed as illustrated in Figure 2(). The SYSTAP's Bigdata [47] is used for RDF data storage consisting of the services repository, WordNet ontology repository, and dependency graph repository. The data stored in these system's repositories were represented in a form of RDF and OWL, which were managed through the Jena API [48].

### 6.2. The Run-Time Subsystem

For the run-time subsystem of the system architecture, the service customers' application (http://202.28.94.50/wscomposition) was also developed as illustrated in Figures 3(a) and 3(b). The user interface for service customers creates a form for searching services through input and output keywords, as shown in Figure 3(a). Then, the results of the WS composition engine are returned to the user and exported as a WS executable description, such as in an OWL-S format, as shown in Figure 3(b).

## 7. System Evaluation and Discussion

This section presents evaluation of the developed WS composition system to verify that the proposed graph-based semantic WS composition approach is appropriate in various application domains. We created a dataset from the WS dataset collected by Zhang et al. [49], the real services published in public registries such as WebserviceX (http://www.webservicex.net/new/Home/Index), and the services repository (http://www.service-repository.com/). These services were categorized into six application domains (healthcare, tourism, business, education, multimedia, and geography). The experiment was designed to measure the system using two metrics, the execution time measurement and the correctness measurement, as presented in the following subsections. The last subsection discusses the contributions of the proposed system and makes a comparison with other approaches.

### 7.1. Execution-Time Measurement

To evaluate the system in terms of execution time, the dataset is divided into five clusters varied by the number of operations of WS from 10 to 1000. The experiment measures the execution times for graph preparation in the management-time processes consisting of parameter preparation (*T_PP_*), parameter matching (*T_PM_*), and graph generation (*T_GG_*), as well as the execution time of the WS composition (*T_WC_*) in the run-time process, as illustrated in Table 5.

In the graph preparation process, the most important observation is that the execution time of the parameter matching process (*T_PM_*) increases significantly with the number of WS because the number of parameter pairs used in finding the semantic similarity (*exact match* and *subsume match*) increased significantly. We also observed that the execution time of WS composition slightly increased with the number of WS because the generated WS dependency graph (arcs and vertices) can create semantic linking between output and input parameters, which can be used to find the generated paths of nonredundant WS composition. The evaluation results show the important of designing two subsystems (i.e., management time and run time) to reduce the execution time of the semantic matchmaking during the run time of the WS composition.

### 7.2. Correctness Measurement

In measuring the correctness of the WS composition system, we set up the experiment for each domain through simulated queries to observe the nonredundant composition results. The correctness is measured using the percentages of precision (2), recall (3), and F-measure (4) metrics with consideration of three numbers: the true positive (TP), which refers to the number of relevant (composite) services to a query; the false positive (FP), which refers to the number of irrelevant services to a query; and the false negative (FN), which refers to the number of relevant services to a query that are not retrieved by the composition system.

(2) $$\begin{array}{c} precision=\left(\frac{TP}{TP+FP}\right)\times 100, \end{array}$$

(3) $$\begin{array}{c} recall=\left(\frac{TP}{TP+FN}\right)\times 100, \end{array}$$

(4) $$\begin{array}{c} F‐measure=\left(2\times \frac{precision\times recall}{precision+recall}\right)\times 100. \end{array}$$

As shown in Table 6, the overall F-measure score (considered as the mean of accuracy of the proposed WS composition system) was 93.49%. The overall exactness indicated by the precision score was 96.76%, whereas the overall completeness of the WS search presented by the recall was 90.43%. Most importantly, the occurrence of errors (*FP* and *FN*) within the search results depended entirely on the correctness of the WS dependency graph generated by the graph preparation. The errors depend on certain keywords extracted from the WS parameters (such as abbreviated or misspelled words) that did not exist in the WordNet repository. Thus, the parameter matching process cannot complete the semantic matchmaking process to connect these parameters, and therefore, it is unable to correctly generate the dependency graph of related services.

### 7.3. Contributions and Comparison with Other Approaches

The main contributions of the paper are to propose (1) the dependency graph preparation technique based on rule reasoning methods of semantic web technologies and (2) the nonredundant WS composition technique based on graph search algorithm. Focusing on these contributions, our work is different from other approaches chosen from the literature review, as described as follows.

In the context of graph-based WS composition, Hashemian and Mavaddat [35] proposed an approach that involves the use of graph search algorithm for finding compositions of WS. However, they did not present the approach for preparing dependency graph of WS which is the most important for potential graph generation. Yue et al. [17] proposed the graph-based approach for WS composition with the graph construction approach which can automatically find the graphical model of related atomic services. However, the matching process for calculating the association degree between atomic services of graph construction approach is based on syntactic consideration. Their proposed approach still lacks to support the semantic matchmaking for solving the semantic conflict problems of WS.

In the context of scalable WS composition, Kwon and Lee [30] proposed a scalable and efficient WS composition approach based on the link index of WS represented in relational database system. Their proposed algorithm can find the nonredundant compositions of WS through indexing of related services. Although, the approach can support the semantic matching of web services' parameters by mapping them to the concepts of the same domain ontology, the examples and experiments presented in the paper did not show the solution of semantic conflicts of parameters of WS.

As for our previous work [40], we proposed the WS annotation model which focused on coping with some kinds of WS's parameter conflict, such as the naming conflict, generalization conflict, and aggregation conflict. Moreover, our previous work defined mapping rules through the Semantic Web Rule Language (SWRL) to transform the healthcare data retrieved from different health organizations. The WS annotation model is represented by adopting an OWL-S which also supports the WS composition model expression. However, this early model did not present an approach of automatic WS composition. The process of constructing the composite process services was done manually by the administrator. In another previous work [41], we aimed to resolve the data-level conflicts of distributed local ontologies extracted from heterogeneous EHR systems. We proposed the Semantic Bridge Ontology to generate mapping rules used to transform local ontology instances into common ontology instances to generate linked-patient data. However, this work did not mention the approach of automatic WS composition.

Our approach in this paper aims to propose the graph-based semantic WS composition techniques which differ from existing researches in the following two main aspects.

1. Dependency graph preparation: In this paper, the graph preparation approach has been proposed to construct the semantic relationships of input and output parameters of the atomic process. This process aims to resolve two types of semantic conflicts of parameters (exact match and subsume match) and provides rule-based reasoning approach to construct the dependency graph model of WS automatically.
2. Nonredundant WS composition technique: This paper presents a graph search algorithm by utilizing the forward search and backward search technique to discovery potential WS paths. We have also proposed an algorithm to eliminate redundant WS from potential WS paths and generate the nonredundant compositions of WS which can be exported to standard executable languages and further used in creating the WS invocation.

## 8. Conclusion and Future Work

In this paper, we presented a semantic WS composition and searching system, which is divided into two subsystems: the management-time subsystem and run-time subsystem. The management-time subsystem delivers dependency graph preparation capable of supporting flexible semantic matches of WS parameters to automatically build the dependency graph of related services. The run-time subsystem creates the WS composition based on a graph-search technique that can efficiently find the most satisfactory results of nonredundant WS compositions. The main contribution of the proposed WS composition system is the production of a technique with which to prepare the WS dependency graph based on the rule reasoning technique of the Semantic Web technologies. The semantic similarity of the WS parameters pairs was identified using both quantitative and qualitative degrees (i.e., similarity score, coefficient, and matching classes), which resulted in the WS dependency graph generation required for reducing the complexity in the WS composition process. Consequently, the system efficiently performs the nonredundant WS composition using a graph search algorithm. Our system was evaluated in the real-world setting of WS within the healthcare domain in the context of locating and invoking the consequential data retrieval services from different electronic health record systems. Additionally, we further evaluated the system in other WS domains, including tourism, business, education, multimedia, and geography, to ensure that the proposed approach performs independently in application domains. However, we recognize two directions for system improvement in future research, as presented in the following paragraphs.

In the aspect of semantic matching within the dependency graph preparation approach, this paper presents only two types of linguistic-based semantic matching rules, the exact match and subsume match, for calculating the similarity score between parameters of the services. To improve this approach in future research, we plan to enhance the system by adding the rules for other semantic similarity strategies, such as statistics-based strategies and natural language processing.

The WS composition approach presented in this paper is based on the graph search methodology *Breadth First Search*. Future improvements to this approach might include the addition of other graph search algorithms to the WS composition engine. Moreover, introducing the nonfunctional capability *Quality of Service* into the engine could possibly enrich the features, making the system more promising for WS composition and searching.

## Figures and Tables

**Figure 12:**
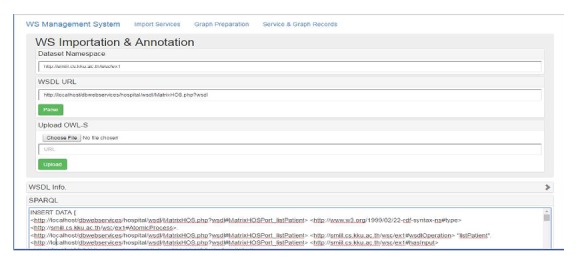
Administrator's WS importation interface.

**Figure 13:**
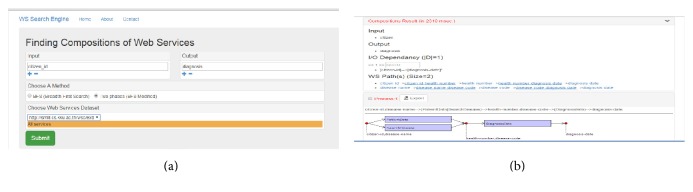
Example of service customer's user interfaces: (a) WS search form and (b) composition results.

**Algorithm 1:**
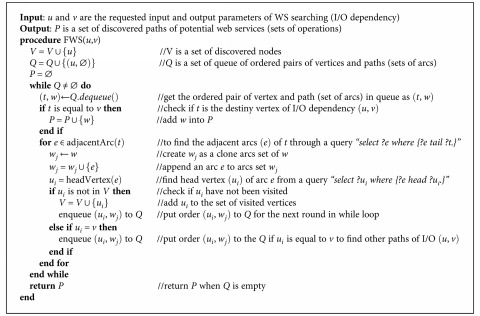
Forward search.

**Algorithm 2:**
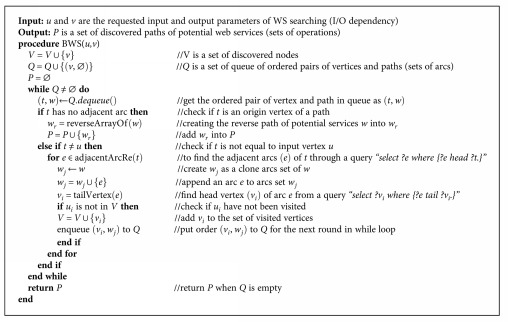
Backward search.

**Algorithm 3:**
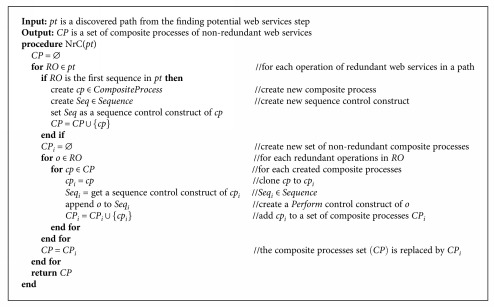
Nonredundant composition.

**Table 1:** Example of web services annotation and queries.

Service	Input	Output	Description
*PatientInfo* (*PI*)	*citizen-id*	*given-name*, *family-name*, *health-number*, *hospital-code*, *district-code*, *professional-id*	This service is offered by a major hospital and performs a query of patient records from a database. Inputting the person's id generates a patient profile including the given and family name, health number, and address, as well as the id of the professional caring for the patient.
*PatientData* (*PD*)	*citizen-id*	*given-name*, *family-name*, *health-number*, *hospital-code, district-code*, *professional-id*	This service is offered by a minor hospital or health care unit, similar to *PatientInfo*.
*PhysicianInfo* (*PHI*)	*physician-id*	*physician-name*	The local service produces the physician record query, which returns a general profile of the physician.
*DiseaseSearch* (*DS*)	*disease-name*	*disease-code*	The local service produces the disease record query, which returns the ICD10 code of the disease from a portion of the disease's name.
*DiagnosisInfo* (*DXI*)	*disorder-code*, *health-number*	*diagnosis-date*	This local service produces the diagnosis record query, which returns the effective date of the specific ICD10 code diagnostic to the specific patient's health number.
*OrganizationInfo* (*OI*)	*organizationCode*	*organization-name*	This governmental service produces an organization record query, which requires an organization code as input and returns a general profile of the organization.
*DistrictInfo* (*DI*)	*district-code*	*district-name*	The governmental service produces a district record query, which returns a profile of a district area through the input district code.
*ZipCodeInfo* (*ZI*)	*CodeOfTerritory*	*zip-code*	The public service produces a postal code query, which returns a postal code of a district area from an input district code.
*Query 1* (*q1*)	*citizen-id*	*health-number*, *organization-name*, *district-name*, *zip-code*	This service query, through the input of a citizen-id number, returns the patient's profile, including health number, name of the hospital of the patient (organization name), and patient address (district name and zip code).
*Query 2* (*q2*)	*citizen-id*	*diagnosis-date*	The requested service returns the effective date of diagnosis through the specific citizen-id of a patient.

**Table 2:** Semantic matching problems definitions.

Matching type	Definition	Examples
Exact match	Let *ap*_*c*_ and *ap*_*k*_ be operations of WS. Let *o*_*c*_ be an output of *ap*_*c*_ and *i*_*k*_ be an input of *ap*_*k*_. If the *o_c_* and *i_k_* are semantically equivalent, denoted by *o*_*c*_≅*i*_*k*_, then the matching of *o*_*c*_ and *i*_*k*_ is an exact match, and *ap*_*c*_ and *ap*_*k*_ can be defined as the sequential processes in the composition.	As shown in Table 1, the output *district-code* of operation *PI* and *PD* contains the keywords {“district”, “code”}, and the input *CodeOfTerritory* of operation *ZI* contains the keywords {“Code”, “Territory”}. Because the keyword “district” ≅ “territory”, and “code” ≅ “code”, the *district-code* and *CodeOfTerritory* are defined as semantically equivalent parameters. Thus, the *PI* (or *PD*) and *ZI* can be defined as sequential processes in the composition.
Subsume match	Let *ap*_*c*_ and *ap*_*k*_ be operations of WS. Let *o*_*c*_ be an output of *ap*_*c*_ and *i*_*k*_ be an input of *ap*_*k*_. If the *o_c_* is more generic than *i_k_*, denoted by *i*_*k*_⊆*o*_*c*_, *o*_*c*_ is a hypernym word and *i*_*k*_ is a hyponym word. We call the *o*_*c*_ and *i*_*k*_ has a subsumption relation or *is-a* relationship. The matching of *o*_*c*_ and *i*_*k*_ is a subsume match, and *ap*_*c*_ and *ap*_*k*_ can be defined as sequential processes in the composition.	The output *hospital-code* of operation *PI* and *PD* contains the keywords {“hospital”, “code”}, and the input *organizationCode* of operation *OI* contains the keywords {“organization”, “code”}. Because the keyword “hospital” is more specific than the keyword “organization”, denoted by “hospital” ⊆ “Organization”, the “hospital” is a hyponym word and the “organization” is a hypernym word. The *hospital-code* and *organizationCode* are defined as subsume match, and the *PI* (or *PD*) and *OI* can be defined as the sequential processes in the composition.
Fail	Let *ap*_*c*_ and *ap*_*k*_ be operations of WS, *o*_*c*_ be an output of *ap*_*c*_, and *i*_*k*_ be an input of *ap*_*k*_. If the *o*_*c*_ and *i*_*k*_ cannot be matched as exact or subsume, these parameters are defined as fail match. The *ap*_*c*_ and *ap*_*k*_ cannot be defined as sequential processes in the composition.	The output *district-name* of *DI* and the input *organization-Code* of *OI* have no semantic similarity; thus, the *DI* and *OI* cannot be defined as sequential processes in the composition.

**Table 3:** Filtering result of the parameter matches instances.

Parameters (*p*_*cm*_, *p*_*kn*_)	Matching instances	Similarity of keywords (*p*)	*q*	*r*	*Co_jac_*	Filtering
*district-code*, *CodeOfTerritory*	*E-district-code-CodeOfTerritory*	2 *(sim-district-territory and sim-code-code)*	0	0	1.0	Keep *E-district-code-CodeOfTerritory*
*hospital-code*, *organizationCode*	*SS-hospital-code-organizationCode* *E-hospital-code-organizationCode*	2 *(sim-hospital- organization and sim-code-code)*	0	0	1.0	Keep *SS-hospital-code-organizationCode* Remove *E-hospital-code-organizationCode*
*district-code*, *organizationCode*	*E-district-code-organizationCode*	1 *(sim-code-code)*	1 {*district*}	1 {*organization*}	0.33	Remove *E-district-code-organizationCode*

**Table 4:** Dependency graph generation rule implemented with SPARQL.

SPARQL rule	Description
INSERT { ?arc rdf:type wse:Arc. ?i1 rdf:type wse:Vertex. ?i2 rdf:type wse:Vertex. ?arc wse:tail ?i1. ?arc wse:head ?i2. ?arc wse:hasOperation ?s1. } WHERE{ ?s1 rdf:type wse:AtomicProcess. ?s1 wse:hasInput ?i1. ?s1 wse:hasOutput ?o1. ?s2 rdf:type wse:AtomicProcess. ?s2 wse:hasInput ?i2. ?s2 wse:hasOutput ?o2. ?m rdf:type wse:SemanticMatching. ?m wse:sourceParameter ?o1. ?m wse:targetParameter ?i2. BIND(IRI(CONCAT(STRBEFORE(STR(?s1),“#”),“#”, STRAFTER(STR(?i1),“#”),“-”, STRAFTER(STR(?i2),“#”))) AS? arc). }	For all atomic processes (*?s*), the input (*?i*) and output (*?o*) of *?s*is expressed by triples, (*?s*, wse:hasInput, *?i*) and (*?s*, wse:hasOutput, *?o*), respectively. Let *?i1* and *?o1* refer to the input and output parameters of atomic process *?s1*, and let *?i2* and *?o2* refer to the input and output parameters of atomic process *?s2*. If there exists matching instance *?m* that maps parameters from *?o1* to *?i2*, then *?arc* is created as the arc of WS dependency graph. *?i1* and *?i2* are expressed as vertices of a graph via triples (*?i1*, rdf:type, wse:Vertex) and (*?i2*, rdf:type, wse:Vertex) and are constructed as the tail and head of *?arc* via triples (*?arc*, wse:tail, *?i1*) and (*?arc*, wse:head, *?i2*). Finally, atomic process *?s1* is added into the redundant operation set of *?arc* via a triple (*?arc*, wse:hasOperation *?s1*).

**Table 5:** Time measurements of graph preparation and WS composition varied by the number of WS.

Number of WS	Number of parameters	*T_PP_* (millisec.)	*T_PM_* (millisec.)	Number of matches	*T_GG_* (millisec.)	Number of arcs/vertices	*T_WC_* (millisec.)
10	13	284	686	20	68	18/13	1151
50	125	1028	27,205	140	1239	1204/125	1477
100	253	1722	42,238	285	22,402	3258/253	1446
500	1074	2539	738,074	764	35,550	4931/452	1160
1000	2411	3919	1,495,853	1506	1,068,746	30,006/2411	2251

**Table 6:** Correctness measurement of WS composition grouped by application domains.

Domain	Number of WS	Number of query	TP	FP	FN	Precision	Recall	F-measure
Healthcare	90	24	220	10	30	95.65%	88.00%	91.66%
Business	26	20	183	1	15	99.45%	92.42%	95.81%
Tourism	144	25	229	11	25	95.41%	90.01%	92.71%
Education	29	18	165	9	22	94.82%	88.23%	91.41%
Multimedia	50	15	137	0	11	100.0%	92.56%	96.14%
Geography	105	22	201	7	17	96.63%	92.20%	94.36%
Overall	444	124	1135	38	120	96.76%	90.43%	93.49%
